# Metformin attenuates lung ischemia-reperfusion injury and necroptosis through AMPK pathway in type 2 diabetic recipient rats

**DOI:** 10.1186/s12890-024-03056-z

**Published:** 2024-05-14

**Authors:** Tianhua Liu, Hong Wei, Lijuan Zhang, Can Ma, Yuting Wei, Tao Jiang, Wenzhi Li

**Affiliations:** 1 Department of Anesthesiology, Hei Long Jiang Province Key Laboratory of Research on Anesthesiology and Critical Care Medicine, Second Affiliated Hospital of Harbin Medical University, NO.246, Xuefu Road, Nangang District, Harbin, Heilongjiang Province 150081 China; 2 Department of Anesthesiology, Sixth Affiliated Hospital of Harbin Medical University, Harbin, China

**Keywords:** Metformin, Type 2 diabetes mellitus, Lung ischemia-reperfusion injury, Necroptosis, AMPK

## Abstract

**Background:**

Diabetes mellitus (DM) can aggravate lung ischemia-reperfusion (I/R) injury and is a significant risk factor for recipient mortality after lung transplantation. Metformin protects against I/R injury in a variety of organs. However, the effect of metformin on diabetic lung I/R injury remains unclear. Therefore, this study aimed to observe the effect and mechanism of metformin on lung I/R injury following lung transplantation in type 2 diabetic rats.

**Methods:**

Sprague–Dawley rats were randomly divided into the following six groups: the control + sham group (CS group), the control + I/R group (CIR group), the DM + sham group (DS group), the DM + I/R group (DIR group), the DM + I/R + metformin group (DIRM group) and the DM + I/R + metformin + Compound C group (DIRMC group). Control and diabetic rats underwent the sham operation or left lung transplantation operation. Lung function, alveolar capillary permeability, inflammatory response, oxidative stress, necroptosis and the p-AMPK/AMPK ratio were determined after 24 h of reperfusion.

**Results:**

Compared with the CIR group, the DIR group exhibited decreased lung function, increased alveolar capillary permeability, inflammatory responses, oxidative stress and necroptosis, but decreased the p-AMPK/AMPK ratio. Metformin improved the function of lung grafts, decreased alveolar capillary permeability, inflammatory responses, oxidative stress and necroptosis, and increased the p-AMPK/AMPK ratio. In contrast, the protective effects of metformin were abrogated by Compound C.

**Conclusions:**

Metformin attenuates lung I/R injury and necroptosis through AMPK pathway in type 2 diabetic lung transplant recipient rats.

**Supplementary Information:**

The online version contains supplementary material available at 10.1186/s12890-024-03056-z.

## Background

Lung transplantation is the most effective treatment for many end-stage lung diseases, such as chronic obstructive pulmonary disease, bronchiectasis, and primary pulmonary hypertension [1]. Lung ischemia-reperfusion (I/R) injury is an inevitable process in lung transplantation and the main cause of primary graft dysfunction, which is associated with the high morbidity and mortality rates in recipients [2]. An unexpectedly high prevalence of diabetes mellitus (DM) and prediabetes was observed in patients awaiting lung transplantation [3]. Of note, DM is a significant risk factor for mortality 1 and 5 years after lung transplantation [4]. Our previous study showed that DM aggravated lung I/R-induced oxidative stress, inflammation, apoptosis and mitochondrial dysfunction [5].

Necroptosis is a newly discovered programmed cell death process. In contrast to apoptosis, necroptosis not only induces cell death but also causes excessive inflammatory damage, and is mediated by the activation of receptor-interacting protein kinase 1 (RIPK1), RIPK3 and mixed lineage kinase domain-like protein (MLKL) [6]. Recent studies have revealed that lung transplantation-associated I/R injury may be related to necroptosis [7, 8]. The RIPK1 inhibitor necrostatin-1 decreases the number of necrotic cells and improves graft function [8]. Hyperglycemia is a condition in which cells are extraordinarily susceptible to necroptosis, and promotes necroptosis in the brain in response to hypoxia-ischemia insult [9]. However, few studies have revealed the importance of necroptosis in lung I/R injury under diabetic conditions.

AMP-activated protein kinase (AMPK) is an important cellular energy sensor that is activated by an increased AMP/ATP ratio and regulates energy homeostasis [10]. AMPK activation can improve mitochondrial function, decrease inflammatory response and oxidative stress [11]. Necroptosis is a pro-inflammatory mode of cell death which is closely related to the depletion of intracellular ATP. Previous studies showed that AMPK is activated under necroptosis induction, and, in turn, AMPK-mediated phosphorylation of Parkin negatively regulates necroptosis to generate a feedback loop that controls necroptosis [12]. However, the effect of AMPK on necroptosis in diabetic lung I/R injury and mechanism remain unclear.

Metformin is widely used as a first-line antidiabetic drug. Two large-scale clinical trials have reported that metformin significantly improves vascular function and reduces any diabetes-related endpoints and all-cause mortality [13, 14]. The beneficial effects of metformin may be mediated through AMPK activation. AMPK activation protects against I/R injury in multiple organs [11, 15–17]. Previous studies demonstrated that metformin attenuated ventilator-induced [18] and lipopolysaccharide-induced lung injury [19, 20]. However, the effect of metformin on lung I/R injury under type 2 diabetic conditions and the mechanism remain unknown.

Therefore, the aims of this study were to determine whether metformin treatment could alleviate diabetic lung I/R injury and necroptosis in an AMPK dependent manner.

## Methods

### Animals

Adult male Sprague–Dawley rats (weighing 200–250 g; 8–9 weeks of age) were purchased from the Animal Center of the Second Affiliated Hospital of Harbin Medical University. The rats were housed in a temperature-controlled room with a 12 h light-dark cycle and free access to a high-fat diet or standard laboratory chow and water before the experiment. The animal experiment procedures were approved by the Institutional Animal Care and Use Committee of Second Affiliated Hospital of Harbin Medical University.

### Type 2 diabetic rat model

The high-fat diet-fed streptozotocin-induced type 2 diabetic rat model is a well-characterized diabetic model [5]. Briefly, the rats were fed high-fat food containing 2.5% cholesterol, 5% sesame oil, 15% lard, 20% sucrose, and 57.5% normal chow for 6 weeks followed by streptozotocin (35 mg/kg) injection intraperitoneally. Then, the rats were continuously fed high-fat food. The rats with fasting plasma glucose levels greater than 11.1 mmol/L 72 h after streptozotocin injection were considered diabetic. Standard laboratory chow-fed rats were used as nondiabetic controls.

### Lung transplantation model

Orthotopic left lung transplantation was performed using a cuff technique as described previously [5]. The donor rats were anesthetized via sevoflurane inhalation, intubated with a 16-gauge cannula through tracheostomy and ventilated with 40% oxygen at a tidal volume of 10 ml/kg with 2 cmH_2_O positive end-expiratory pressure. Five minutes after intravenous injection of sodium heparin (500 U/kg), the donor rats underwent median sternotomy, and the lungs were flushed with 20 ml of saline at 4 °C at a pressure of 20 cm H_2_O through the pulmonary artery. Then, the donor lungs were harvested, attached to cuff tubes and preserved at 4 °C for 60 min.

The recipient rats were anesthetized, orally intubated and ventilated with similar settings as the donors. After a left thoracotomy, the left lung artery, vein and bronchus were anastomosed with those of the donor lung by the cuff technique. During lung transplantation, tidal volumes were decreased to 6 mL/kg and restored to 10 mL/kg immediately after reperfusion. After lung transplantation, all recipient rats received analgesia with 0.25% ropivacaine by local infiltration anesthesia. After 24 h of reperfusion, recipient rats were sacrificed by exsanguination, and samples were collected.

### Experimental groups

The rats were randomly divided into 6 groups (*n* = 8): control + sham (CS) group, DM + sham (DS) group, control + I/R (CIR) group, DM + I/R (DIR) group, DM + I/R + metformin (DIRM) group and DM + I/R + metformin + Compound C (DIRMC) group. In all of the I/R groups, the donor rats were nondiabetic rats, and recipient rats were type 2 diabetic rats except those in the CIR group. Rats in the sham group were only subjected to left thoracotomy without transplantation. Metformin was administered intravenously at a dose of 200 mg/kg immediately after reperfusion. Compound C, a selective AMPK inhibitor, was administered intraperitoneally at a dose of 5 mg/kg 20 min before reperfusion. The doses of metformin and Compound C were selected based on previous studies [17, 21].

### Glucose level monitoring

Blood glucose levels were monitored before transplantation (T0) and 12 h (T1) and 24 h (T2) after reperfusion.

### Blood gas analysis

Arterial blood was drawn through the femoral artery from the recipients and measured 24 h after reperfusion. The PaO_2_/FiO_2_ ratio was calculated.

### Measurement of static compliance of lung grafts

Median sternotomy was performed immediately after sacrifice. Then, the lung grafts were connected to a homemade apparatus to obtain static pressure-volume curves. The lung volume was determined by increasing the airway pressure to 30 cm H_2_O and then decreasing it to 0 cm H_2_O with 1 min of stabilization in 5 cm H_2_O stepwise intervals. The lung volumes were corrected for gas compression in the apparatus.

### Histopathologic analysis

The lung grafts were fixed in 4% paraformaldehyde and embedded in paraffin. The 5 μm sections were prepared and stained with hematoxylin and eosin. The extent of lung injury was evaluated blindly using a histological scoring system as described previously [22].

### Assessment of mitochondrial morphology

Approximately 1 mm^3^ of the lung graft was collected and fixed in 2.5% glutaraldehyde to observe mitochondrial morphology in type II alveolar epithelial cells by transmission electron microscopy.

### Measurement of the wet-to-dry weight (W/D) ratio of lung grafts

The wet weight of the upper part of the lung graft was measured immediately after harvest, and then the specimen was placed in an 80 °C oven for 72 h to measure the dry weight. The lung W/D ratio was calculated.

### Measurement of total protein concentrations in bronchoalveolar lavage fluid (BALF)

Total protein concentrations in BALF were estimated using a BCA protein assay kit according to the manufacturer’s instructions.

### Immunofluorescence analysis

After dewaxing, dehydrating and blocking, lung paraffin sections were incubated with rabbit polyclonal anti-ZO-1 (1:80, 21773-1-AP, Proteintech) and mouse monoclonal anti-occludin (1:80, 66378-1-Ig, Proteintech) antibodies overnight at 4 °C and then conjugated with goat anti-rabbit IgG (1:50, ZF-0516, ZSGB-BIO) and goat anti-mouse IgG (1:50, ZF-0512, ZSGB-BIO) fluorescent secondary antibodies for 30 min at 37 ℃. Images were acquired using a fluorescence microscope. The fluorescence intensities of ZO-1 and occludin were quantified by ImageJ software.

### Measurement of inflammatory cytokine levels

The interleukin (IL)-1β (No.JM-01454R1, Jingmei Biotechnology), IL-6 (No.JM-01597R1, Jingmei Biotechnology), tumor necrosis factor-α (TNFα) (No.JM-01587R1, Jingmei Biotechnology) and IL-10 (No.JM-01602R1, Jingmei Biotechnology) kits were used to measure the levels of IL-1β, IL-6, TNFα and IL-10 in serum and BALF. After the lower part of the lung graft was homogenized, myeloperoxidase (MPO) activity was determined using a commercially available assay kit (No. A044-1-1, Nanjing Jiancheng Bioengineering Institute).

### Measurement of oxidative stress marker levels

The lower part of the lung graft was homogenized and centrifuged to obtain the supernatant. Malondialdehyde (MDA) (No. A003-1-2, Nanjing Jiancheng Bioengineering Institute), superoxide dismutase (SOD) (No. A001-3-2, Nanjing Jiancheng Bioengineering Institute) and total anti-oxidative capability (T-AOC) (No. A015-2-1, Nanjing Jiancheng Bioengineering Institute) kits were used to measure the MDA level, SOD activity and T-AOC activity in transplanted lung tissues.

### Analysis of reactive oxygen species (ROS) levels

The lower part of the fresh lung graft was embedded in an optimal cutting temperature compound and quick-frozen in liquid nitrogen. The 8 μm sections were prepared and incubated with dihydroethidium at 37 ℃ for 30 min in the dark. Images were taken with a fluorescence microscope. The fluorescence intensity was quantified by ImageJ software.

### Measurement of cell necrosis marker levels

The high mobility group box-1 (HMGB-1) kit (No.JM-01893R1, Jingmei Biotechnology) was used to measure the level of HMGB-1 in serum and BALF.

### Western blot analysis

The extracted protein samples were separated by SDS-PAGE and transferred onto PVDF membranes. After being blocked, the membranes were incubated with rabbit polyclonal anti-AMPK (1:1000, AF6423, Affinity Biosciences), rabbit polyclonal anti-p-AMPK (1:1000, AF3423, Affinity Biosciences), rabbit polyclonal anti-RIPK1 (1:1000, AF7877, Affinity Biosciences), rabbit polyclonal anti-RIPK3 (1:1000, AF7942, Affinity Biosciences), rabbit polyclonal anti-MLKL (1:1000, DF7412, Affinity Biosciences), rabbit polyclonal anti-HMGB-1 (1:1000, AF7020, Affinity Biosciences) and rabbit polyclonal anti-β-actin (1:1000, AF7018, Affinity Biosciences) antibodies at 4 ℃ overnight. Then, the membranes were incubated with the horseradish peroxidase-conjugated secondary antibodies (1:1000; SA00001-2, Proteintech) for 2 h. Protein band intensity was quantified with ImageJ software.

### Statistical analysis

All data are expressed as the mean ± standard deviation (SD). Differences among groups were analyzed by one-way analysis of variance, followed by the Tukey test for multiple comparisons. All statistical analyses were performed using GraphPad Prism 9.0 software. A value of *P* < 0.05 was considered statistically significant.

## Results

### Metformin did not reduce blood glucose levels

**Fig. 1:**
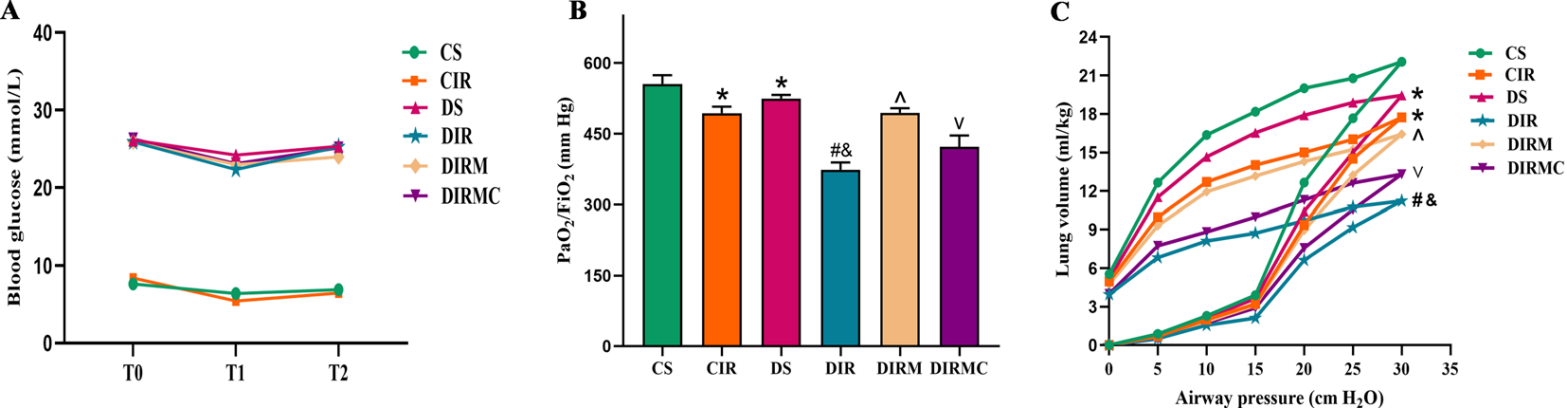
Metformin improved the function of lung grafts in type 2 diabetic lung transplant recipient rats. (**A**) Blood glucose level (*n* = 8). T0-T2 represented the following time points: baseline, 12 and 24 h after reperfusion. Data were presented as the mean values, and standard deviation bars were omitted for clarity. (**B**) PaO_2_/FiO_2_ ratio (*n* = 8). (**C**) Static pressure-volume curve of lung grafts (*n* = 3). Data were presented as the mean values, and standard deviation bars were omitted for clarity. Compared with CS group, ^*^P<0.05; compared with CIR group, ^#^*P*<0.05; compared with DS group, ^&^*P*<0.05; compared with DIR group, ^^^*P*<0.05; compared with DIRM group, ^v^*P*<0.05

After 12 h and 24 h of reperfusion, there was no significant changes in blood glucose in the DIR group and DIRM group (*P* > 0.05), indicating that metformin did not significantly reduce blood glucose levels in type 2 diabetic recipient rats (Fig. 1A).

### Lung graft function

The PaO_2_/FiO_2_ ratio was decreased in the DS group compared with the CS group (*P* < 0.05). Compared with that in the CIR group, the ratio was significantly reduced in the DIR group (*P* < 0.05). Metformin significantly increased the ratio compared with that in the DIR group (*P* < 0.05). However, the effect of metformin was reversed by Compound C (*P* < 0.05) (Fig. 1B).

At an airway pressure of 30 cm H_2_O, the volumes in the pressure-volume curve in the DS group were reduced compared with those in the CS group (*P* < 0.05). Compared with those in the CIR group, the volumes were decreased in the DIR group (*P* < 0.05). Metformin treatment significantly increased the volumes compared with that in the DIR group (*P* < 0.05). However, this effect of metformin was abolished by Compound C (*P* < 0.05) (Fig. 1C).

### Lung appearance

**Fig. 2:**
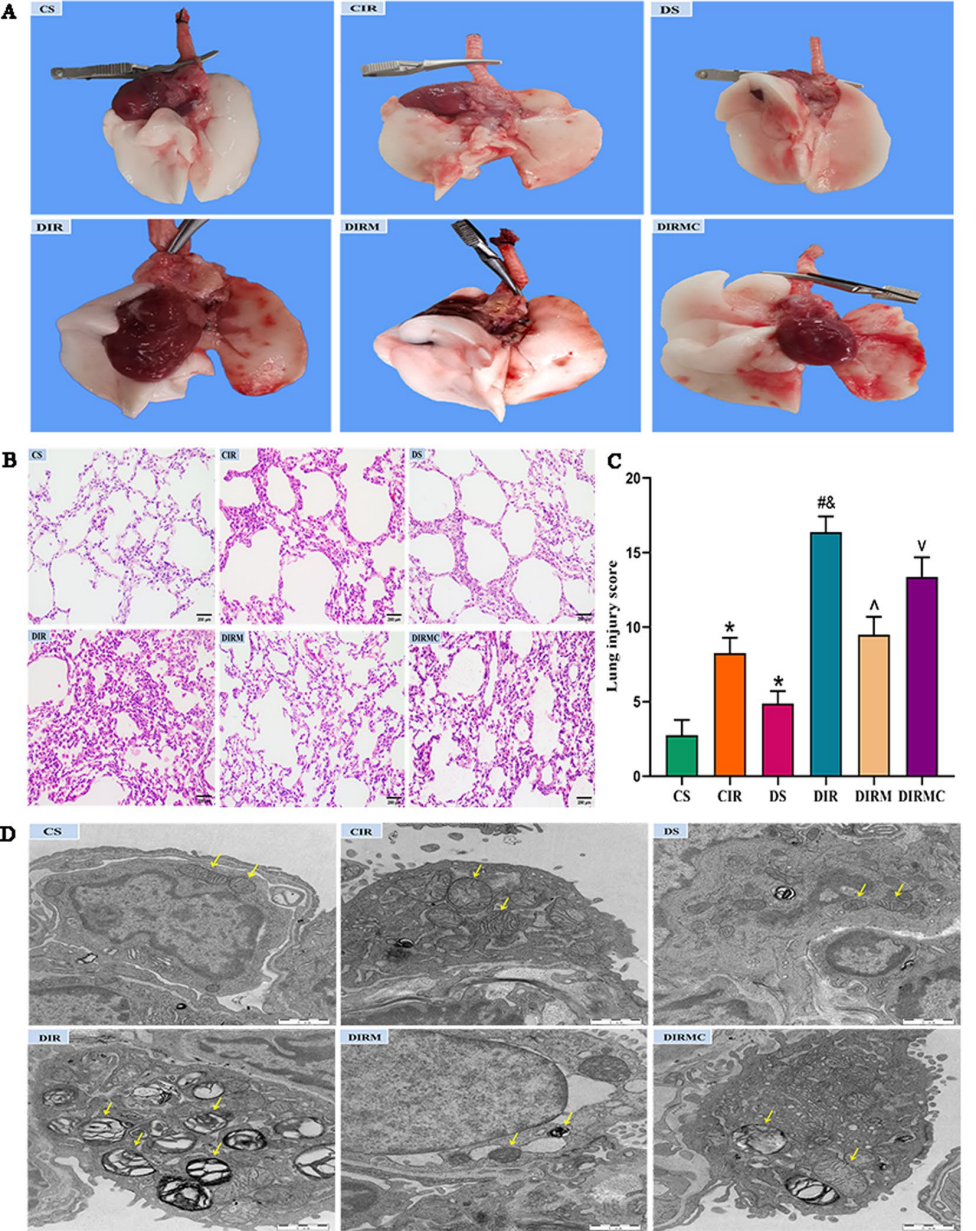
Metformin mitigated the lung I/R-induced damage in type 2 diabetic lung transplant recipient rats. (**A**) Lung appearance. (**B**) HE staining representative images (magnification: ×200, scale bar: 200 μm). (**C**) Lung injury score (*n* = 6). (**D**) Mitochondrial ultrastructure representative images of alveolar type II epithelial cells (magnification: ×20,000, scale bar: 1 μm). As was shown in the picture, the yellow arrows showed the mitochondria. Compared with CS group, ^*^P<0.05; compared with CIR group, ^#^*P*<0.05; compared with DS group, ^&^*P*<0.05; compared with DIR group, ^^^*P*<0.05; compared with DIRM group, ^v^*P*<0.05

After slow perfusion through the pulmonary artery, the whole lung in the CS group was white, smooth, and had good compliance. Most of the transplanted lung was pink, and there was edema and reduced compliance in the CIR group. The lungs in the DS group exhibited a light pink color, were smooth, and had reduced compliance. The transplanted lung was dark red and rough, with severe edema and reduced compliance in the DIR group. Melatonin alleviated transplanted lung injury, but this improvement was attenuated in the DIRMC group (Fig. 2A).

#### Lung graft histology

Histologically, the left lung tissues in the CS group were almost normal. However, the DS group showed epithelial cell injury, thickened basement membranes and inflammatory cell infiltration. The CIR group showed numerous pathological changes including severe interstitial edema, massive inflammatory cell infiltration, diffuse alveolar damage and intra-alveolar hemorrhage. More serious histological changes were present in the DIR group. Metformin ameliorated the histological changes, and Compound C aggravated the histological changes. The lung injury score directly confirmed this result (*P* < 0.05) (Fig. 2B and C).

#### Mitochondrial morphology of lung grafts

Ultrastructural changes in type II alveolar epithelial cells were observed by transmission electron microscopy. The mitochondrial membrane and cristae were almost intact in the CS group. A few mitochondrial cristae were broken in the DS group. Most mitochondria were severely swollen, with vacuoles and no mitochondrial cristae in the CIS group. These morphological alterations were more notable in the DIR group. Treatment with metformin mitigated the changes in mitochondrial morphology. Compound C abolished these effects of metformin (Fig. 2D).

#### Alveolar-capillary permeability of lung grafts

**Fig. 3:**
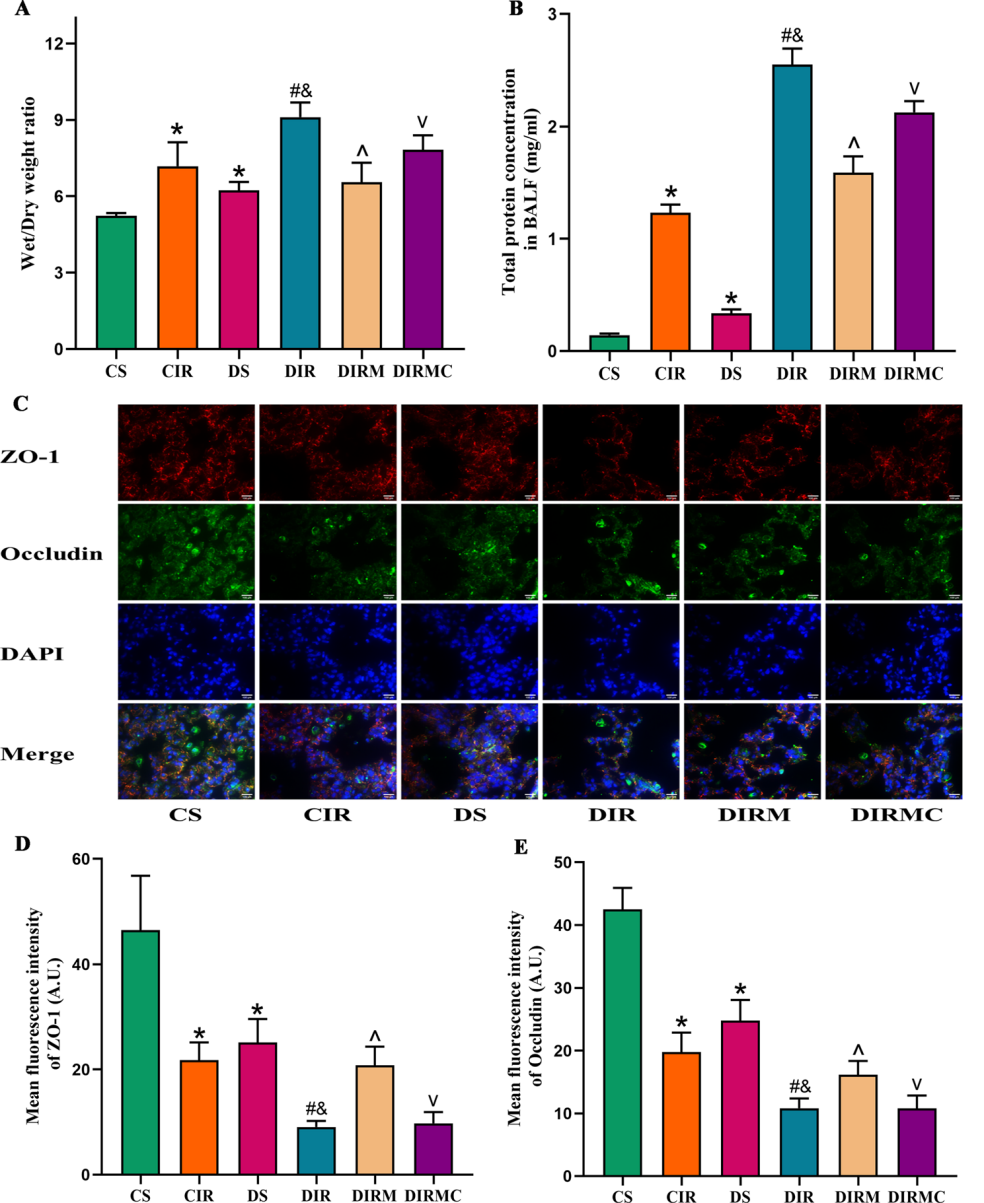
Metformin ameliorated the alveolar-capillary permeability of lung grafts in type 2 diabetic lung transplant recipient rats. (**A**) Wet/Dry ratio (*n* = 8). (**B**) Total protein concentration in BALF (*n* = 4). (**C**) Immunofluorescence staining representative images of tight junction proteins (magnification: ×400, scale bar: 100 μm). As was shown in the picture, ZO-1 was red, occludin was green, and nucleus was blue. (**D**) ZO-1 levels in lung grafts (*n* = 5). (**E**) Occludin levels in lung grafts (*n* = 5). Compared with CS group, ^*^P<0.05; compared with CIR group, ^#^*P*<0.05; compared with DS group, ^&^*P*<0.05; compared with DIR group, ^^^*P*<0.05; compared with DIRM group, ^v^*P*<0.05

Compared with that in the CS group, the W/D ratio was increased in the DS group (*P* < 0.05). The ratio was increased in the DIR group compared with the CIR group (*P* < 0.05). Metformin treatment markedly reduced the ratio compared with that in the DIR group (*P* < 0.05). However, the effect of metformin was reversed by Compound C (*P* < 0.05) (Fig. 3A). Total protein concentrations in BALF exhibited similar changes as the W/D ratio (Fig. 3B). The ZO-1 and occludin expression levels in the lung grafts exhibited the opposite changes as the W/D ratio (Fig. 3C-E).

### Inflammatory responses in the recipients

**Fig. 4:**
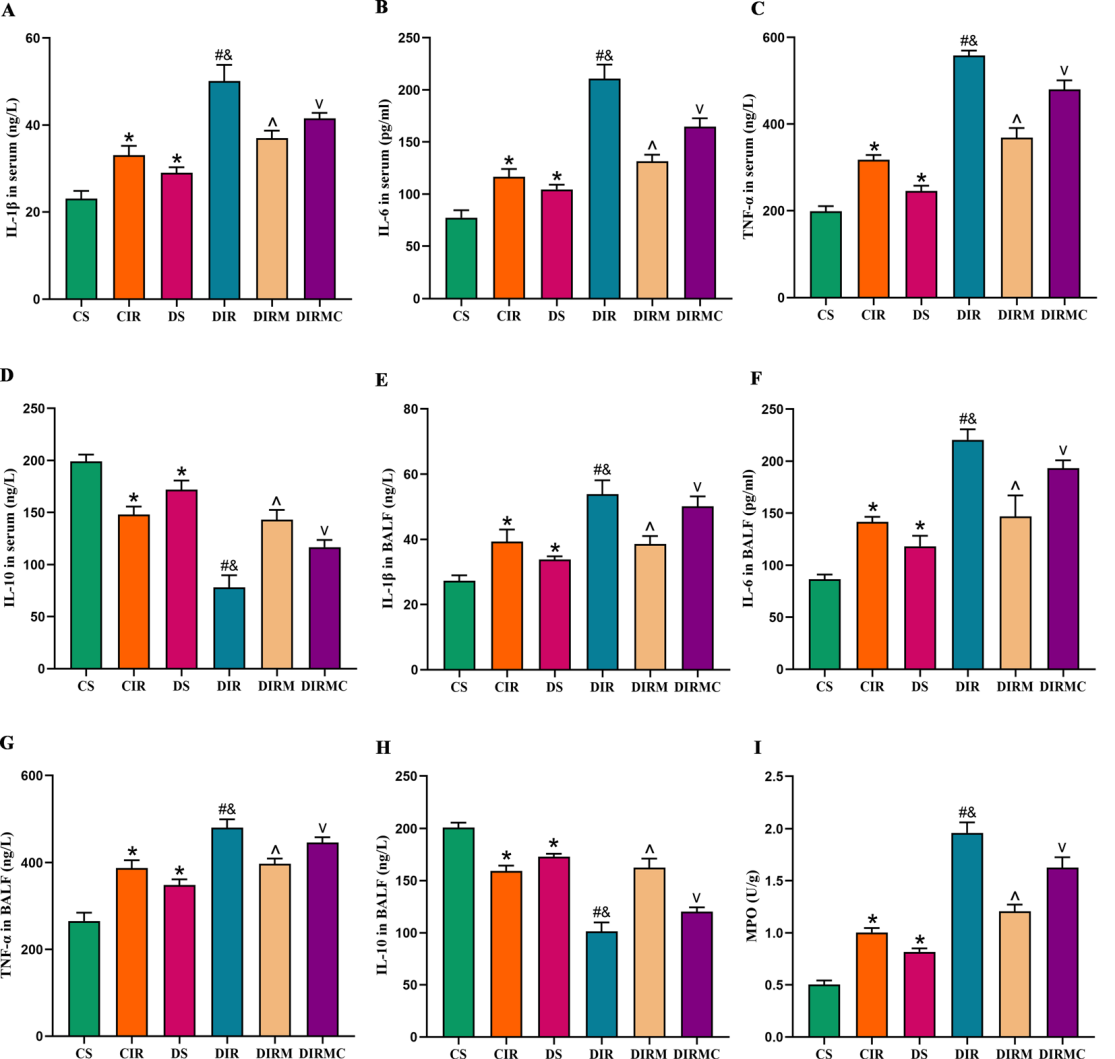
Metformin reduced the inflammatory response in type 2 diabetic lung transplant recipient rats. (**A**) IL-1β concentration in serum (*n* = 8). (**B**) IL-6 concentration in serum (*n* = 8). (**C**) TNF-α concentration in serum (*n* = 8). (**D**) IL-10 concentration in serum (*n* = 8). (**E**) IL-1β concentration in BALF (*n* = 4). (**F**) IL-6 concentration in BALF (*n* = 4). (**G**) TNF-α concentration in BALF (*n* = 4). (H) IL-10 concentration in BALF (*n* = 4). (I) MPO activity in lung grafts (*n* = 6). Compared with CS group, ^*^P<0.05; compared with CIR group, ^#^*P*<0.05; compared with DS group, ^&^*P*<0.05; compared with DIR group, ^^^*P*<0.05; compared with DIRM group, ^v^*P*<0.05

Compared with those in the CS group, serum levels of the pro-inflammatory factors IL-1β, IL-6 and TNF-α were increased in the DS group (*P* < 0.05). These pro-inflammatory factors levels were increased in the DIR group compared with the CIR group (*P* < 0.05). Treatment with metformin dramatically reduced the pro-inflammatory factors levels compared with that in the DIR group (*P* < 0.05). However, the effects of metformin were abolished by Compound C (*P* < 0.05) (Fig. 4A-C). In contrast, the anti-inflammatory factor IL-10 exhibited the opposite changes (Fig. 4D). These inflammatory factors in BALF exhibited similar changes as those in serum (Fig. 4E-H). In addition, MPO activity in lung grafts exhibited similar changes as the pro-inflammatory factors levels (Fig. 4I).

### Oxidative stress in the recipients

**Fig. 5:**
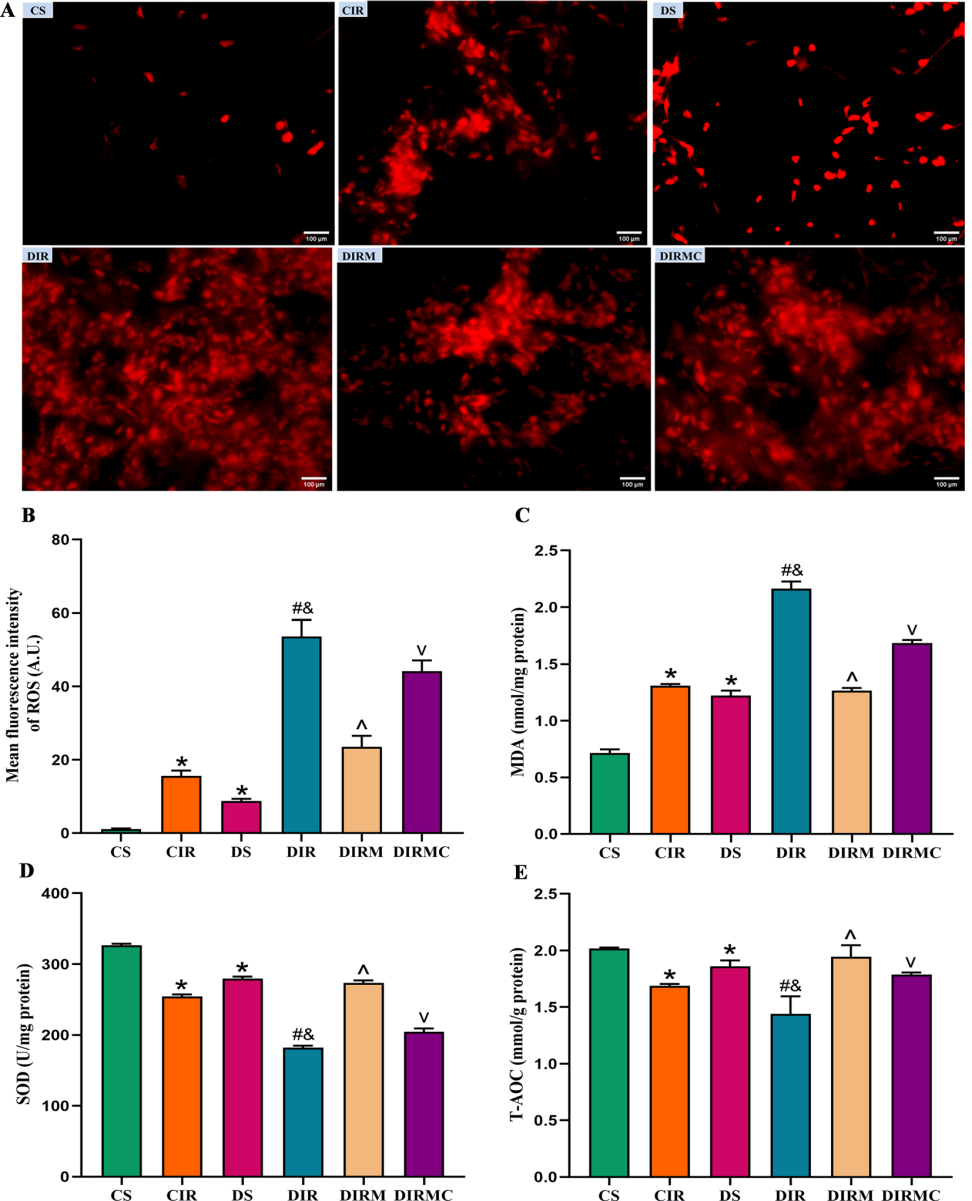
Metformin reduced the oxidative stress in type 2 diabetic lung transplant recipient rats. (**A**) ROS fluorescence staining representative images (magnification: ×400, scale bar: 100 μm). (**B**) ROS levels in lung grafts (*n* = 3). (**C**) MDA levels in lung grafts (*n* = 8). (**D**) SOD activity in lung grafts (*n* = 8). (**E**) T-AOC activity in lung grafts (*n* = 8). Compared with CS group, ^*^P<0.05; compared with CIR group, ^#^*P*<0.05; compared with DS group, ^&^*P*<0.05; compared with DIR group, ^^^*P*<0.05; compared with DIRM group, ^v^*P*<0.05

Compared with that in the CS group, the ROS levels in lung grafts in the DS group were increased (*P* < 0.05). Compared with that in the CIR group, the ROS levels were increased in the DIR group (*P* < 0.05). Metformin treatment dramatically decreased the ROS levels compared with that in the DIR group (*P* < 0.05). Compound C administration inhibited the beneficial effects of metformin (*P* < 0.05) (Fig. 5A and B). Moreover, MDA levels in lung grafts exhibited similar changes as ROS levels (Fig. 5C). In contrast, the changes in SOD and T-AOC activities were the opposite of those of ROS levels (Fig. 5D and E).

### Cell necrosis marker levels in the recipients

**Fig. 6:**
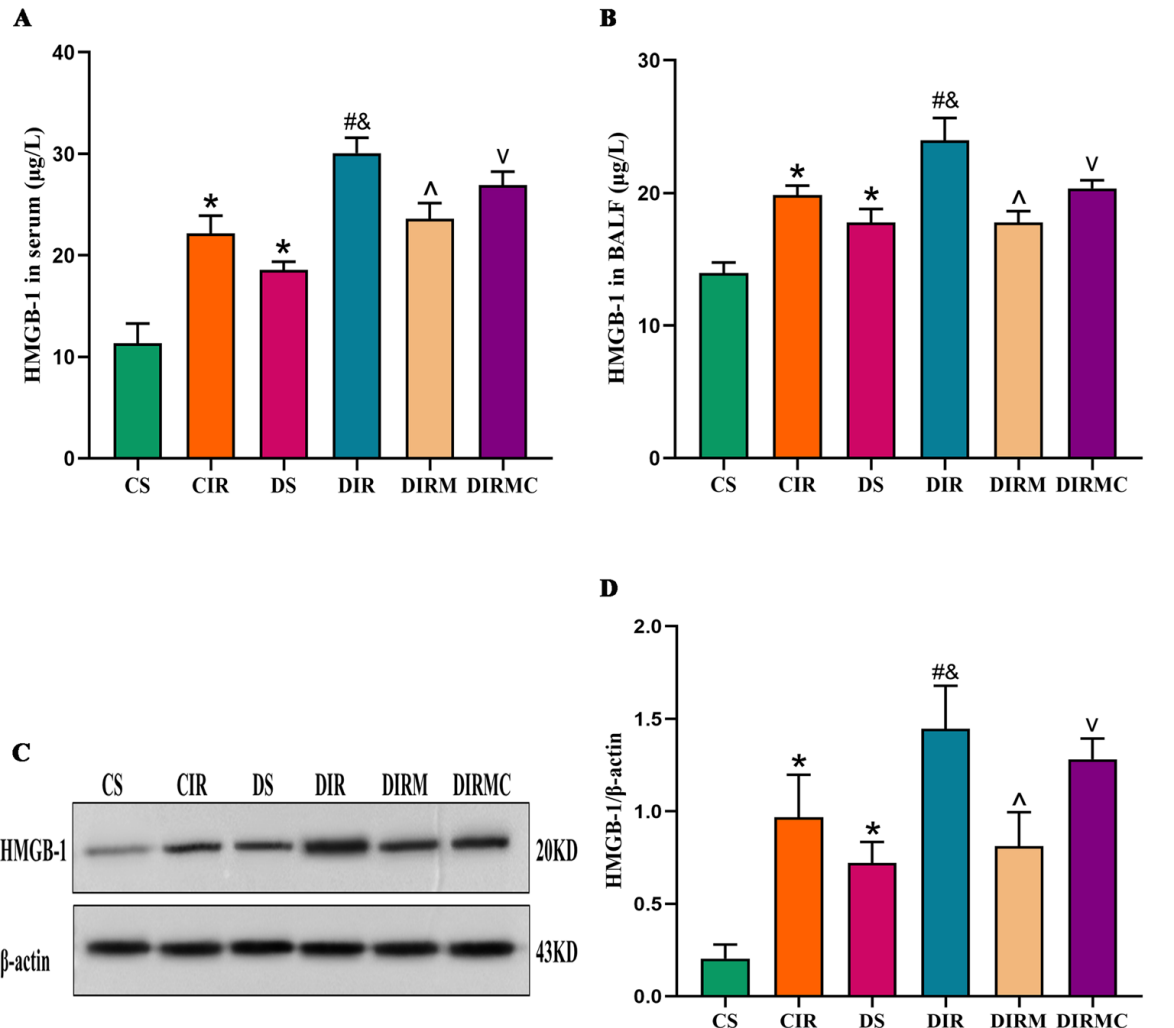
Metformin decreased the HMGB-1 levels in type 2 diabetic lung transplant recipient rats. (**A**) HMGB-1 concentration in serum (*n* = 8). (**B**) HMGB-1 concentration in BALF (*n* = 4). (**C**) HMGB-1 representative western blot image. Full-length blots/gels are presented in Supplementary Fig. 1. (**D**) HMGB-1 expression levels in lung grafts (*n* = 3). Compared with CS group, ^*^P<0.05; compared with CIR group, ^#^*P*<0.05; compared with DS group, ^&^*P*<0.05; compared with DIR group, ^^^*P*<0.05; compared with DIRM group, ^v^*P*<0.05

Compared with those in the CS group, serum HMGB-1 levels in the DS group were increased (*P* < 0.05). Compared with those in the CIR group, HMGB-1 levels were increased in the DIR group (*P* < 0.05). Metformin markedly decreased HMGB-1 levels compared with that in the DIR group (*P* < 0.05), and the effects of metformin were reversed by Compound C (*P* < 0.05) (Fig. 6A). BALF and lung graft HMGB-1 levels showed similar changes as serum HMGB-1 (Fig. 6B-D).

### Expression levels of necroptosis key proteins in lung grafts

**Fig. 7:**
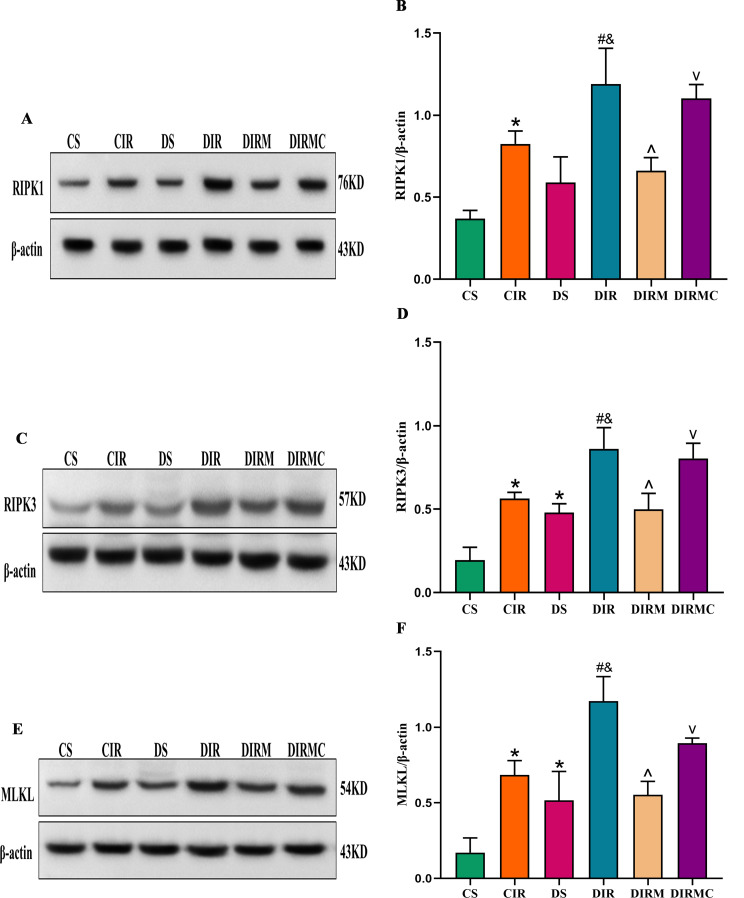
Metformin inhibited the necroptosis in type 2 diabetic lung transplant recipient rats. (**A**) RIPK1 representative western blot image. Full-length blots/gels are presented in Supplementary Fig. 2. (**B**) RIPK1 expression levels in lung grafts (*n* = 3). (C) RIPK3 representative western blot image. Full-length blots/gels are presented in Supplementary Fig. 3. (**D**) RIPK3 expression levels in lung grafts (*n* = 3). (**E**) MLKL representative western blot image. Full-length blots/gels are presented in Supplementary Fig. 4. (**F**) MLKL expression levels in lung grafts (*n* = 3). Compared with CS group, ^*^P<0.05; compared with CIR group, ^#^*P*<0.05; compared with DS group, ^&^*P*<0.05; compared with DIR group, ^^^*P*<0.05; compared with DIRM group, ^v^*P*<0.05

Compared with those in the CS group, the expression levels of RIPK1 in the DS group were increased (*P* > 0.05), and the expression levels of RIPK3 and MLKL were increased (*P* < 0.05). Compared with those in the CIR group, the expression levels of RIPK1, RIPK3 and MLKL in the DIR group were increased (*P* < 0.05). Metformin dramatically decreased the expression levels of RIPK1, RIPK3 and MLKL compared with that in the DIR group (*P* < 0.05), and the effects of metformin were abolished by Compound C (*P* < 0.05) (Fig. 7A-F).

### AMPK signaling pathway-related protein expression levels in lung grafts

**Fig. 8:**
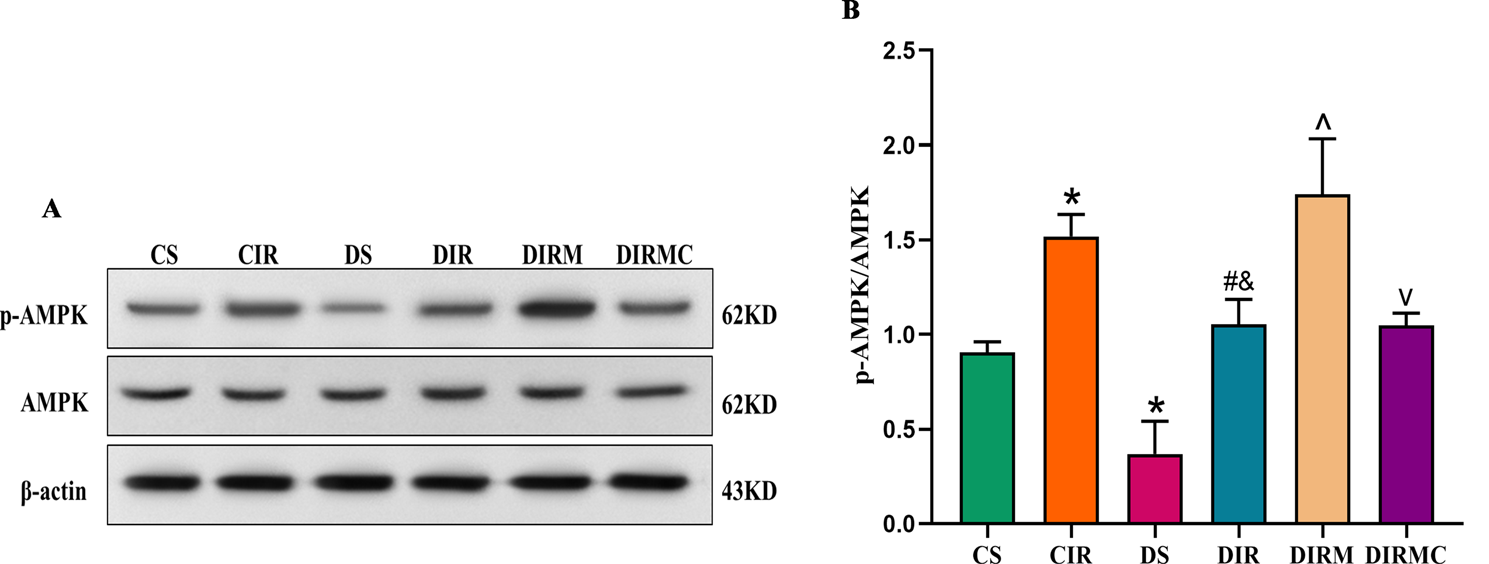
Metformin activated the AMPK signal pathway in type 2 diabetic lung transplant recipient rats. (**A**) AMPK and p-AMPK representative western blot image. Full-length blots/gels are presented in Supplementary Figs. 5 and 6. (**B**) p-AMPK/AMPK ratio in lung grafts (*n* = 3). Compared with CS group, ^*^P<0.05; compared with CIR group, ^#^*P*<0.05; compared with DS group, ^&^*P*<0.05; compared with DIR group, ^^^*P*<0.05; compared with DIRM group, ^v^*P*<0.05

The ratios of p-AMPK/AMPK were decreased in the DS group compared with the CS group (*P* < 0.05). Compared with those in the CIR group, the ratios were reduced in the DIR group (*P* < 0.05). Metformin significantly increased the ratios compared with that in the DIR group (*P* < 0.05). Compound C administration significantly decreased the ratios (*P* < 0.05) (Fig. 8A and B).

## Discussion

The major findings of this study were as follows: i) type 2 DM exacerbated lung I/R injury which was characterized by increases in inflammatory cytokines, oxidative stress markers and necroptosis; ii) metformin ameliorated the inflammatory response, oxidative stress and necroptosis in diabetic lung transplantation recipient rats; and iii) these beneficial effects of metformin were associated with the upregulation of AMPK activation.

Many studies have shown that DM, especially type 2 DM, is associated with lung disorder [23–25]. Persistent hyperglycemia upregulates advanced glycation end products, the polyol pathway and protein kinase C pathway [22, 26]. The combination of these mechanisms eventually contributes to inflammation response, oxidative stress and cell death. DM is a major risk factor for mortality after lung transplantation and directly influences the outcomes of lung transplantation [27]. The mechanisms by which DM increases morbidity and mortality are complex and not yet fully understood.

Necroptosis may be one of the important mechanisms of diabetic lung injury after lung transplantation. A growing number of studies have shown that apoptosis plays a critical role in the loss of lung function in nondiabetic rats that are lung transplantation recipients [28, 29]. However, necroptosis appears to be predominant in diabetic rats that are lung transplantation recipients. In the TNF-α pathways, which is associated with apoptosis and necroptosis, there is some overlap in the initial steps [30, 31]. Both pathways involve the formation of membrane–proximal protein complexes, and then reach a point of divergence, in which different cellular contexts may favor one pathway over the other. Interestingly, hyperglycemia conditions favor necroptosis over apoptosis [32]. Consistent with these findings, our results showed that the levels of necroptosis-related proteins were significantly increased in diabetic rat lung grafts, confirming the occurrence of necroptosis.

In contrast to apoptosis, necroptosis is a significant pathogenic factor in numerous inflammatory diseases [33, 34]. Necroptosis may lead to the loss of plasma membrane integrity and the release of damage-associated molecular patterns (DAMPs) [35]. HMGB-1, a marker of cell necrosis, is a major DAMP, that contributes to inflammatory responses. Extracellular HMGB-1 can activate macrophages, which are important inflammatory cells associated with lung injury. Activated macrophages produce many pro-inflammatory cytokines [36], which further activate macrophages, resulting in a vicious cycle that amplifies the inflammatory response. MPO, an indicator of neutrophil infiltration and accumulation, may be related to pro-inflammatory cytokine production and endothelial and epithelial barrier destruction [37]. In diabetic lung injury, severe inflammatory response was observed [38], which was consistent with our study. In addition, our study also showed that metformin had anti-inflammatory effect, which was also demonstrated by other studies [39, 40]. In our study, metformin effectively suppressed the secretion of IL-1β, IL-6 and TNF-α and reduced HMGB1 protein expression and MPO levels in diabetic rats after lung transplantation, and these effects were abolished by Compound C. These results suggested that metformin could attenuate the inflammatory response in diabetic lung transplantation recipient rats.

Oxidative stress is a key factor that plays an important role in lung I/R injury in diabetes [22]. RIPK3 is a key determinant of necroptosis that can promote excessive ROS production [41, 42]. Excessive production of ROS can lead to lipid peroxidation and damage the membranes of the cell and mitochondria, ultimately causing necroptosis. Therefore, necroptosis and ROS levels may form a vicious cycle that increases oxidative stress and aggravates lung I/R injury. Hence, ROS overload may be the main marker of oxidative damage in the present study. The present findings were consistent with our previous studies which showed that type 2 diabetes mellitus enhanced oxidative stress, which was characterized by increased MDA levels and decreased SOD and T-AOC activities in a rat lung I/R model [4, 22]. Our findings showed that metformin could markedly increase the activities of SOD and T-AOC and reduce the levels of ROS and MDA in diabetic rats. The beneficial effects of metformin were reversed by Compound C. These results indicated that metformin partly restored the balance between oxidation and anti-oxidation in diabetic lung transplantation recipient rats, which was consistent with the findings of previous study [43].

Necroptosis directly causes epithelial and endothelial cell necrosis, leading to alveolar-capillary barrier disruption. In addition, excessive inflammation and oxidative stress contribute to barrier dysfunction. HMGB-1 might contribute to lung endothelial barrier dysfunction and lung vascular hyperpermeability. Inhibiting HMGB-1 could protect against lung endothelial barrier dysfunction [44]. Oxidative stress could induce endothelial and epithelial cell injury, increase vascular permeability and promote the formation of lung edema. It has been reported that inhibiting oxidative stress might be an effective therapeutic option [45]. In the present study, we used the tight junction proteins ZO-1 and occluding to estimate the integrity of the alveolar-capillary barrier. Our results showed that metformin could reverse the I/R-induced downregulation of ZO-1 and occluding expression and alleviate alveolar-capillary permeability and lung edema. Other study also consistently demonstrated that metformin preserved alveolar-capillary barrier and alleviated lung edema [46]. Metformin-mediated barrier protection was abolished by Compound C. These results indicated that metformin prevented the disruption of the alveolar-capillary barrier by inhibiting the inflammation response, oxidative stress and necroptosis in diabetic lung transplantation recipient rats.

To further investigate the mechanism underlying metformin-mediated protection against lung I/R injury in the diabetic state, we investigated the effect of metformin on the AMPK pathway. Many studies have demonstrated that metformin protects against various diseases due to its activation of AMPK, which plays an important role in modulating the inflammatory response and oxidative stress [11]. Metformin amplified the I/R-induced increase in AMPK activity, which was beneficial to the damaged myocardium due to the ability of AMPK to promote ATP generation and ameliorate cardiomyocyte apoptosis [47, 48]. Metformin downregulated the expressions of necroptosis key proteins, suppressed the inflammatory response and improved renal function via the AMPK pathway in MRL/lpr lupus-prone mice [49]. In the present study, our research findings also showed that metformin further increased lung I/R-induced upregulation of AMPK activity, suppressed necroptosis and mitigated lung I/R injury under diabetic conditions. The anti-necroptotic effect of metformin was abrogated by Compound C. These results indicated that metformin significantly ameliorated diabetic lung I/R injury and necroptosis through the AMPK pathway.

There are also some limitations to this study. First, the effect of different concentrations of metformin on diabetic lung I/R injury after transplantation was not explored. Future studies are required to investigate the effects of different concentrations of metformin on diabetic lung I/R injury in order to determine the most appropriate dose. Second, because the prolonged cold ischemia times may trigger necroptosis, we chose relative short cold ischemia times in the present study. But, in the clinical setting, the occurrence of such short cold ischemia times is infrequent. Therefore, in the future study, we will choose a prolonged cold ischemia times to further study. Third, although we found that metformin could protect against necroptosis caused by lung I/R in the diabetic state via the AMPK pathway, how the AMPK pathway influences necroptosis remains unknown. A20, an inhibitor of the NF-κB signaling pathway, may play a pivotal role in the effect of AMPK on necroptosis, which needs to be further investigation. Fourth, this study only conducted experimental exploration on male rat and did not conduct the same research on female rat. Finally, no clinical experimental data were available for this study. Further clinical studies are needed to validate the effects of metformin in humans.

## Conclusions

In summary, our study showed that type 2 diabetes mellitus further aggravated lung I/R-induced inflammatory responses, oxidative stress and necroptosis after transplantation. Metformin treatment could significantly ameliorate lung I/R injury and necroptosis by activating the AMPK pathway, thus improving lung function in diabetic lung transplantation recipient rats.

### Electronic supplementary material

Below is the link to the electronic supplementary material.

Supplementary Material 1

Supplementary Material 2

## Data Availability

The datasets generated in this study are available from the corresponding author upon reasonable request.

## Abbreviations

AMPK: AMP-activated protein kinase
BALF: bronchoalveolar lavage fluid
DAMPs: damage-associated molecular patterns
DM: diabetes mellitus
HMGB-1: high mobility group box-1
I/R: ischemia reperfusion
IL: interleukin
MPO: myeloperoxidase
MDA: malondialdehyde
MLKL: mixed lineage kinase domain-like protein
RIPK1: receptor-interacting protein kinase 1
ROS: reactive oxygen species
SOD: superoxide dismutase
SD: standard deviation
TNFα: tumor necrosis factor-α
T-AOC: total anti-oxidative capability
W/D: wet-to-dry weight ratio
