# Supplementary material for: Metformin attenuates lung ischemia-reperfusion injury and necroptosis through AMPK pathway in type 2 diabetic recipient rats
Source: BMC Pulm Med. 2024 May 14;24:237. doi: 10.1186/s12890-024-03056-z (PMC11094932; doi:10.1186/s12890-024-03056-z)

Original images of Western blot

1. Full-length blot of HMGB-1

CS CIR DS DIR DIRM DIRMC

β-actin 43KD

HMGB-1 20KD

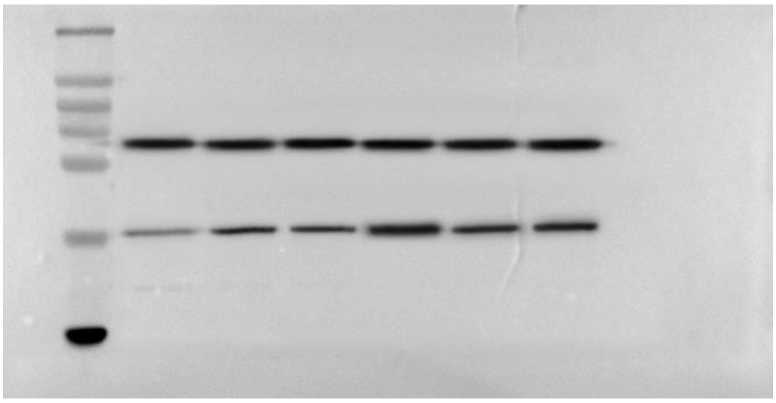

2. Full-length blot of RIPK1

CS CIR DS DIR DIRM DIRMC

RIPK1 76KD

β-actin 43KD

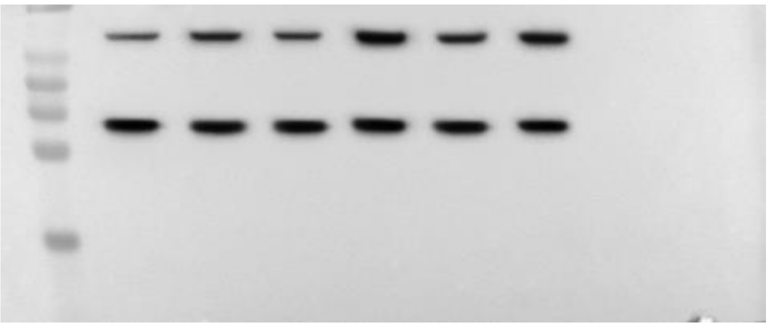

3. Full-length blot of RIPK3

CS CIR DS DIR DIRM DIRMC

RIPK3 57KD

β-actin 43KD

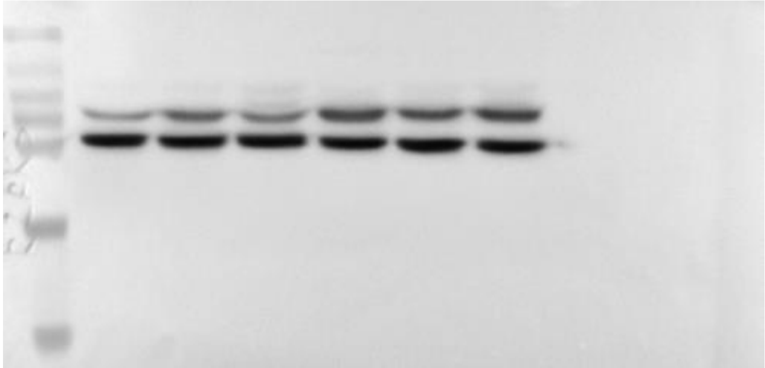

4. Full-length blot of MLKL

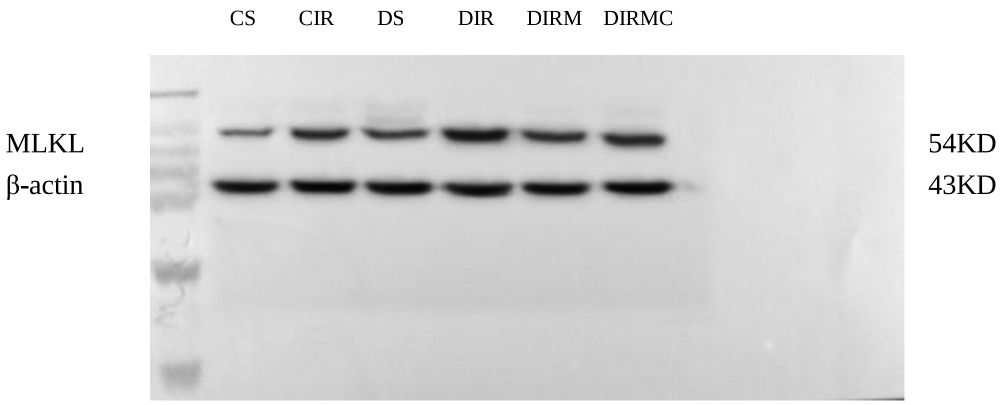

5. Full-length blots of AMPK and p-AMPK

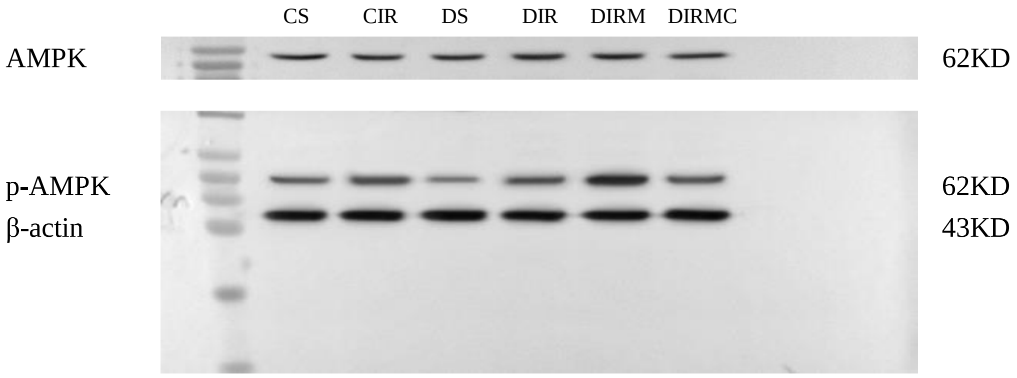

Supplement: Supplementary file 1 — Supplementary Material 1 [file 12890_2024_3056_MOESM1_ESM.pdf]
