# Supplementary material for: Metformin attenuates lung ischemia-reperfusion injury and necroptosis through AMPK pathway in type 2 diabetic recipient rats
Source: BMC Pulm Med. 2024 May 14;24:237. doi: 10.1186/s12890-024-03056-z (PMC11094932; doi:10.1186/s12890-024-03056-z)
Supplement: Supplementary file 2 — Supplementary Material 2 [file 12890_2024_3056_MOESM2_ESM.pdf]

Original images of Western blot are as following:

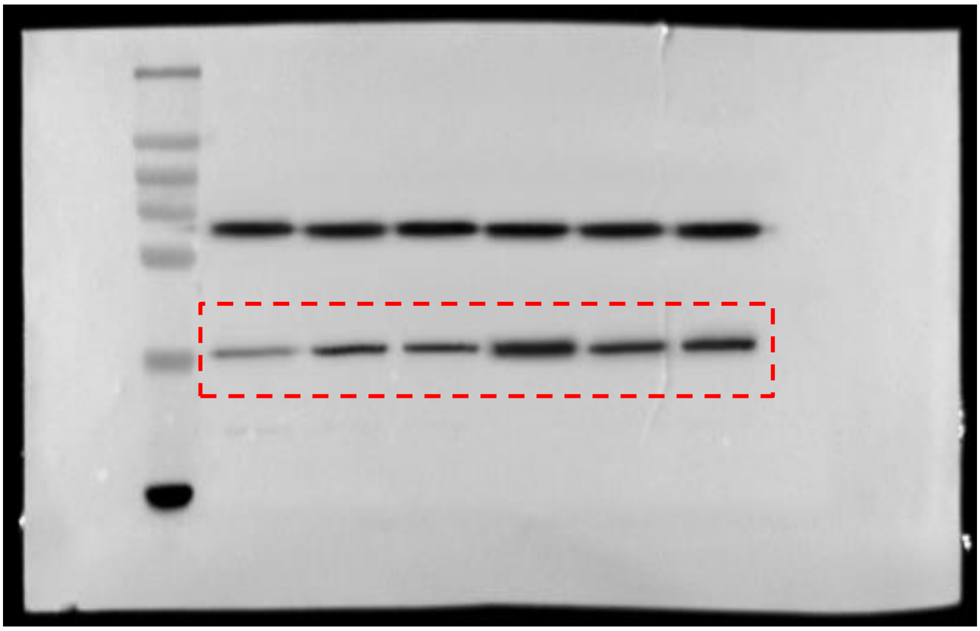

Supplementary Figure 1: Original images of HMGB-1.

The immunoblots below show HMGB-1. The immunoblots above show β-actin. The red rectangle dotted line indicates where they were cropped. Each juxtaposition lane respectively represents CS, CIR, DS, DIR, DIRM and DIRMC group except marker from left to right.

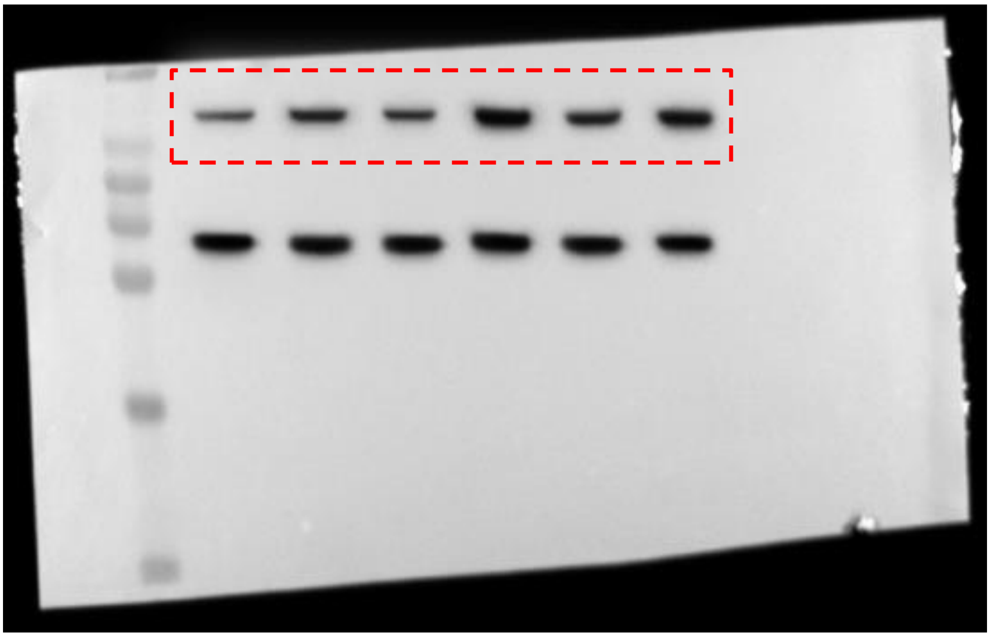

Supplementary Figure 2: Original images of RIPK1.

The immunoblots above show RIPK1. The immunoblots below show β-actin. The red

rectangle dotted line indicates where they were cropped. Each juxtaposition lane respectively represents CS, CIR, DS, DIR, DIRM and DIRMC group except marker from left to right.

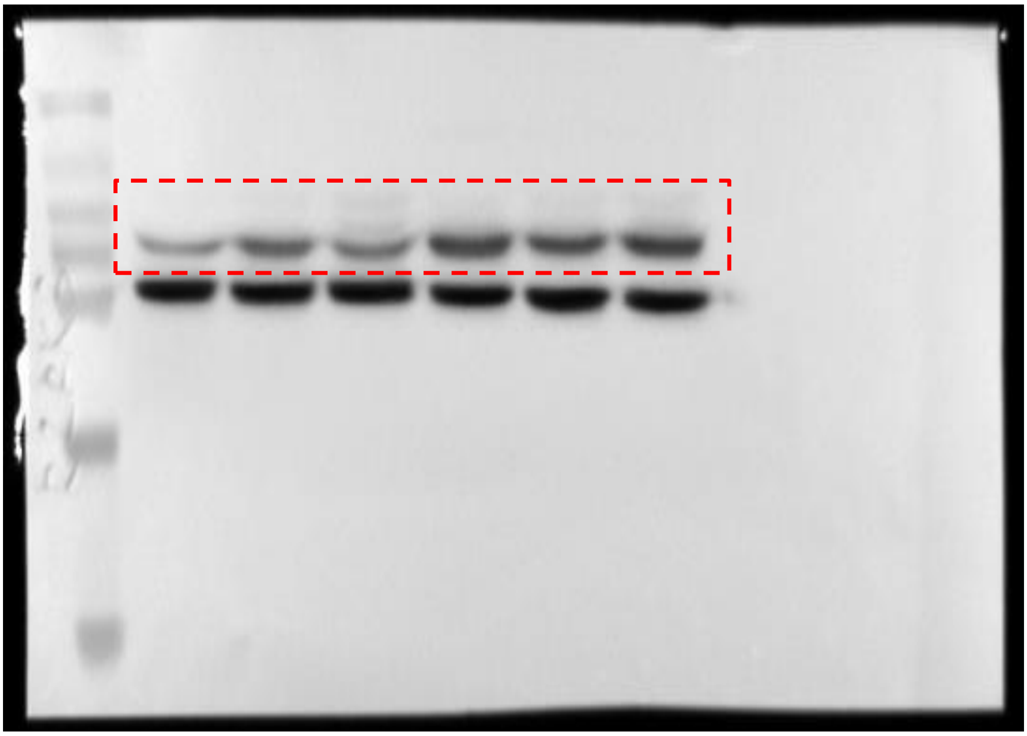

Supplementary Figure 3: Original images of RIPK3.

The immunoblots above show RIPK3. The immunoblots below show β-actin. The red rectangle dotted line indicates where they were cropped. Each juxtaposition lane respectively represents CS, CIR, DS, DIR, DIRM and DIRMC group except marker from left to right.

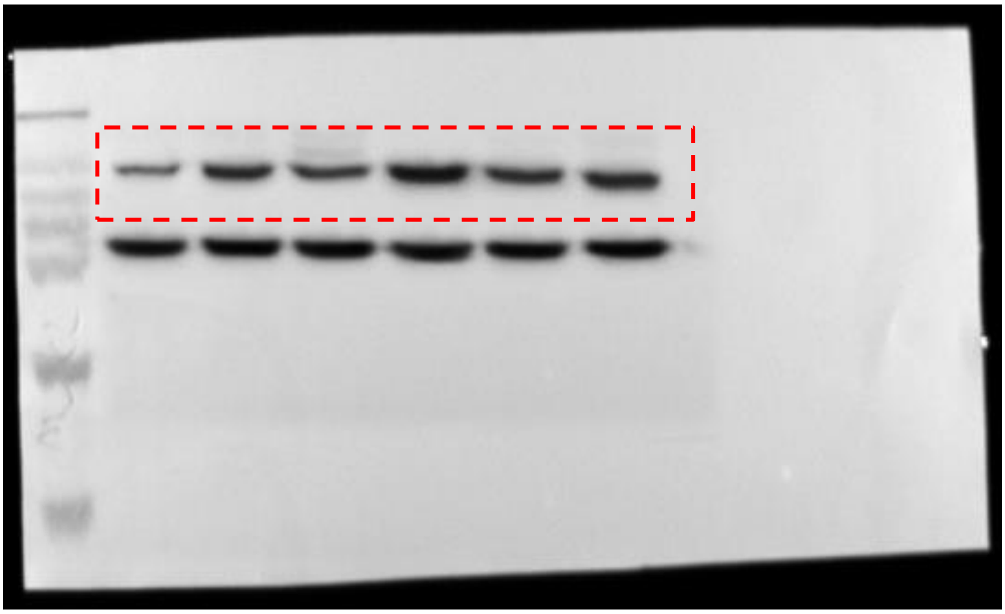

Supplementary Figure 4: Original images of MLKL.

The immunoblots above show MLKL. The immunoblots below show β-actin. The red rectangle dotted line indicates where they were cropped. Each juxtaposition lane respectively represents CS, CIR, DS, DIR, DIRM and DIRMC group except marker from left to right.

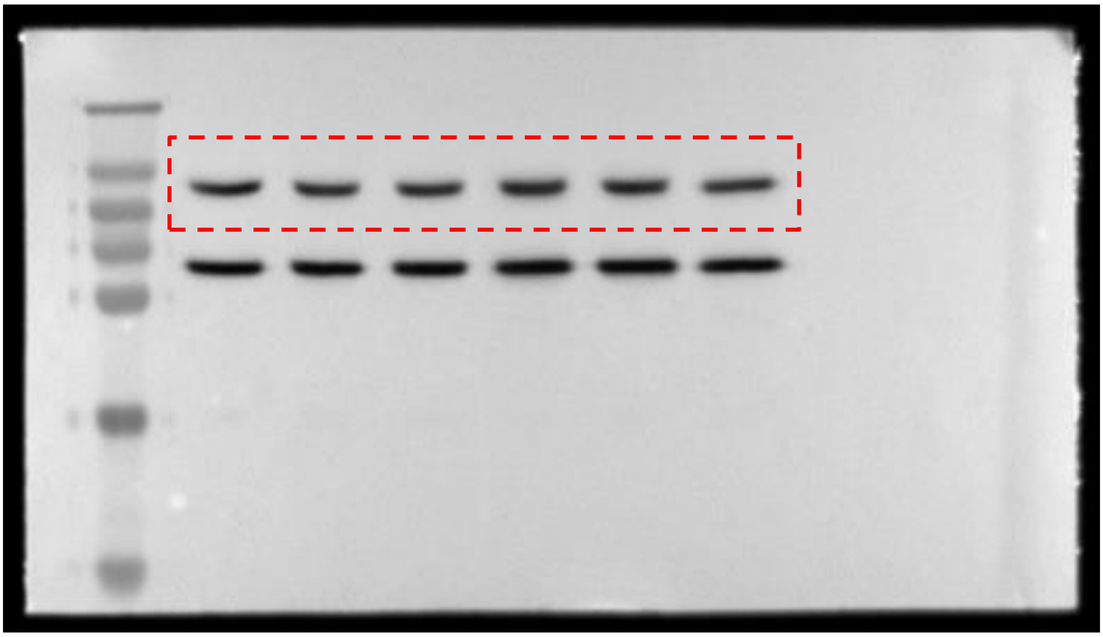

Supplementary Figure 5: Original images of AMPK.

The immunoblots above show AMPK. The immunoblots below show β-actin. The red rectangle dotted line indicates where they were cropped. Each juxtaposition lane respectively represents CS, CIR, DS, DIR, DIRM and DIRMC group except marker

from left to right.

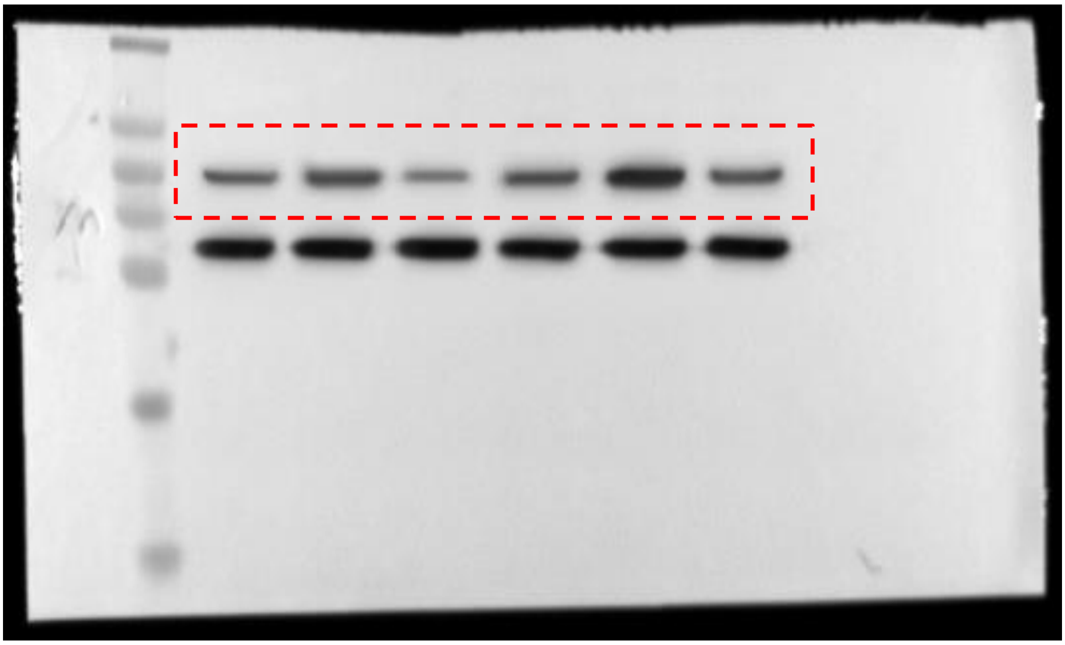

Supplementary Figure 6: Original images of p-AMPK.

The immunoblots above show p-AMPK. The immunoblots below show β-actin. The red rectangle dotted line indicates where they were cropped. Each juxtaposition lane respectively represents CS, CIR, DS, DIR, DIRM and DIRMC group except marker from left to right.

All methods in sufficient detail are as following:

Glucose level monitoring

The tail skin of rats was disinfected with iodophor, the blood was collected by needling the tail vein, and the blood glucose levels were measured by glucose meter. The fasting blood glucose of the recipient rats before operation was measured as the basic value, which was recorded as T0. The blood glucose levels 12 hours after reperfusion was recorded as T1. The blood glucose level 24 hours after reperfusion was measured as T2.

Blood gas analysis

The recipient rats were anesthetized, intubated and ventilated with similar settings as the donors 24 hours after reperfusion. After mechanical ventilation for 5 minutes, 0.2ml of arterial blood was drawn through the femoral artery from the recipients and measured (Rapid Lab 348, Bayer, Medfield, United States). The PaO_2/FiO_2 ratio was calculated.

Measurement of static compliance of lung grafts

Median sternotomy was performed immediately after sacrifice. The thorax was fully unfolded, and the right pulmonary hilum was ligated. Then, the lung grafts were connected to a homemade apparatus through endotracheal tube to obtain static pressure-volume curves. The lung volume was determined by increasing the airway pressure to 30 cm H_2O and then decreasing it to 0 cm H_2O with 1 minute of stabilization in 5 cm H_2O stepwise intervals. The lung volumes were corrected for gas compression in the apparatus.

Histopathologic analysis

a. The middle of the left lung was immediately fixed in 4% paraformaldehyde at 4˚C for 48h.

b. After 48h, the lung tissues were soaked in 70%, 80%, 90% and 95% alcohol for 30 minutes respectively for dehydration. The lung tissues were soaked in anhydrous ethanol - xylene mixture, xylene I and xylene II for 30 minutes respectively.

c. The tissues were soaked in paraffin I and paraffin II for 30 minutes respectively and embedded in paraffin. Then, 5μm paraffin sections were prepared.

d. After being dewaxed, sections were stained with hematoxylin and eosin for 30 min

at room temperature.

e. All images were acquired with a Nikon Eclipse 80i microscope (Nikon Corporation, Tokyo, Japan).

f. The degree of the lung injury was assessed with a histologic study score using the following criteria: (1) neutrophil infiltration, (2) airway epithelial cell damage, (3) interstitial edema, (4) hyaline membrane formation, and (5) hemorrhage. Each criterion was scored on a semi-quantitative scale of 0 to 4 as follows: normal=0, minimal change=1, mild change=2, moderate change=3, and severe change=4.

Assessment of mitochondrial morphology

a. Approximately 1 mm^3 of the lung graft was collected and fixed with 2.5% glutaraldehyde at 4℃ overnight and then post-fixed with fixative B at 4℃ for 2 hours.

b. Samples were dehydrated in a stepwise acetone at 4℃ and embedded in 812 Epon resin.

c. After resin polymerization at 70℃ for 2 days, 70 nm section was cut by an UC6 ultra-microtome (Leica Microsystems) and stained with uranyl acetate and lead citrate before being analyzed in a H-7650 (Hitachi, Tokyo, Japan) operated at 80 kV.

Measurement of the wet-to-dry weight ratio of lung grafts

a. The wet weight of the upper part of the lung graft was measured immediately after harvest.

b. The specimen was placed in an 80 ℃ oven for 72 h to measure the dry weight.

c. The lung W/D ratio was calculated.

Measurement of total protein concentrations in BALF

The total protein concentrations in BALF were measured according to the manufacturer's instruction using enhanced BCA protein assay kit (No. P0010, Beyotime).

a. 1.2 ml of BSA Preparation Solution was added into one tube of BSA Standard (30 mg BSA) provided in this kit to make a 25 mg/ml BSA standard solution after fully dissolving. Then, 0.5 mg/ml BSA standard solution was prepared.

b. According to the number of assays, an appropriate amount of BCA Working Solution was prepared by mixing BCA reagent A and BCA reagent B at a ratio of 50:1.

c. 0, 1, 2, 4, 8, 12, 16, and 20 μl of the 0.5mg/ml BSA standard solution were pipetted to the standard wells of the 96-well plate, and the sample preparation buffer was added to a final volume of 20 μl per well, which is equivalent to 0, 0.025, 0.05, 0.1, 0.2, 0.3, 0.4, and 0.5 mg/ml, respectively.

d. An appropriate volume of sample was added to the sample wells of the 96-well plate. If the sample is less than 20 μl, the sample preparation buffer was added to a final volume of 20 μl per well. Record the sample volume used.

e. 200 μl of BCA Working Solution were added to each well and incubated at 37 ℃ for 30 minutes.

f. The absorbance at 562nm was measured with a microplate reader.

g. The protein concentration of the sample was calculated based on the standard curve and the sample volume used.

Immunofluorescence analysis

a. The paraffin sections were dehydrated in xylene I (15 minutes), xylene II (15 minutes), anhydrous ethanol I (5 minutes), anhydrous ethanol II (5 minutes), 85% alcohol (5 minutes), 75% alcohol (5 minutes).

b. The sections were placed in citric acid-sodium citrate antigen repair solution at high temperature for 10 minutes.

c. The sections was covered with goat serum and incubated at room temperature for 30 minutes.

d. Lung paraffin sections were incubated with rabbit polyclonal anti-ZO-1 (1:80, 21773-1-AP, Proteintech) and mouse monoclonal anti-occludin (1:80, 66378-1-Ig, Proteintech) antibodies overnight at 4 ℃ and then conjugated with fluorescent secondary antibodies at 37 °C for 30 minutes.

e. The sections were counterstained with DAPI, and then a cover slip was placed on slides.

f. Images were taken with a fluorescence inverted microscope (Olympus IX71, Japan).

Measurement of inflammatory cytokine levels

The levels of IL-1β, IL-6, TNFα and IL-10 in serum and BALF were quantified using enzyme-linked immunosorbent assay kits (Jingmei Biotechnology, China). These inflammatory cytokine levels were measured according to the manufacturer’s instructions. The measure methods in detail are as follows:

a. The concentration gradient standard products were prepared according to the instructions.

b. 50μl standard products were added into the standard well on the 96-wells Elisa

plate.

c. 40μl sample diluent was added into the sample well, and then 10μl sample was added into the sample well.

d. The 96-wells Elisa plate was placed in a 37℃ incubator for 30 minutes in the dark.

e. The 96-wells in plate were washed fully with scrubbing solution. Then, 50μl of enzyme-labeled reagent was added to each well except the blank well.

f. After the 96-wells in plate were incubated and washed again, 50μl of color developing agent A and B were added into each well. Then, the 96-wells Elisa plate was placed in a 37℃ incubator for 10 minutes in the dark.

g. 50μl termination solution was added into each well. The absorbance at 450nm of each well was measured with a microplate reader.

h. The inflammatory cytokine levels of the sample were calculated based on the standard curve.

Measurement of MPO activity

MPO activity was determined using MPO assay kit (No.A044-1-1, Nanjing Jiancheng Bioengineering Institute) according to the manufacturer's instructions.

a. The lung tissue is weighed and placed into a 5 ml homogenate tube. 19 times the volume of reagent II is added into the homogenate tube according to mass (g)-volume (ml) ratio of 1:19. The lung tissue is cut to small pieces and fully homogenized in ice water bath to make 5% tissue homogenate.

b. Operation procedure is performed according to the instructions in the MPO assay kit. After the operation procedure is completed, the spectrophotometer is used to

measure the absorbance of each tube at 460nm.

c. Formulas:

MPO activity (u/g) = (OD_{Sample}−$OD_{Contrast}$)/(11.3×Sample weight)

Measurement of oxidative stress marker levels

MDA levels, SOD activity and T-AOC activity in transplanted lung tissues were measured using the respective kits (Nanjing Jiancheng Bioengineering Institute, China) according to the manufacturer's instructions. The measure methods of oxidative stress markers in detail are as follows:

MDA levels were measured using MDA assay kit (No. A003-1-2, Nanjing Jiancheng Bioengineering Institute).

a. The lung tissue is weighed and placed into a 5 ml homogenate tube. 9 times the volume of normal saline is added into the homogenate tube according to mass (g)-volume (ml) ratio of 1:9. The lung tissue is cut to small pieces and fully homogenized in ice water bath to make 10% tissue homogenate. The 10% tissue homogenate was centrifuged at 4℃, 2,500 r/min for 15 minutes and the supernatant was taken for determination.

b. Operation procedure is performed according to the instructions in the MDA assay kit. After the operation procedure is completed, the spectrophotometer is used to measure the absorbance of blank tube, standard tube, contrast tube and sample tube at 532nm.

c. Formulas:

MDA content (nmol/mg protein)=(OD_{Sample}-$OD_{Contrast}$)/($OD_{Standard}$-OD_{Blank})×Standard

concentration÷Protein concentration in sample to assay

SOD activity was measured using SOD assay kit (No. A001-3-2, Nanjing Jiancheng Bioengineering Institute).

a. The lung tissue is weighed and placed into a 5 ml homogenate tube. 9 times the volume of normal saline is added into the homogenate tube according to mass (g)-volume(ml) ratio of 1:9. The lung tissue is cut to small pieces and fully homogenized in ice water bath to make 10% tissue homogenate. The 10% tissue homogenate was centrifuged at 4℃, 2,500 r/min for 10 minutes and the supernatant was taken for determination.

b. After sample preparation above, protein concentration in sample is measured by BCA method.

c. Operation procedure is performed according to the instructions in the SOD assay kit. After the operation procedure is completed, the enzyme-labeled instrument is used to measure the absorbance of contrast well, contrast blank well, sample blank well and sample well at 450nm.

d. Formulas:

SOD inhibition percentage = $[(A_{Contrast}-A_{Contrast\ blank}) - (A_{Sample}-A_{Sample\ blank})] / (A_{Contrast}-A_{Contrast\ blank})\times 100\%$

SOD activity (U/mg protein) = SOD inhibition percentage÷50%×Reaction system dilution times×Protein concentration in sample to assay

T-AOC activity was measured using T-AOC assay kit (No. A015-2-1, Nanjing Jiancheng Bioengineering Institute).

a. The lung tissue is weighed and placed into a 5 ml homogenate tube. 9 times the volume of normal saline is added into the homogenate tube according to mass (g)-volume(ml) ratio of 1:9. The lung tissue is cut to small pieces and fully homogenized in ice water bath to make 10% tissue homogenate. The 10% tissue homogenate was centrifuged at 4℃, 12,000 r/min for 5 minutes and the supernatant was taken for determination.

b. After sample preparation above, protein concentration in sample is measured by BCA method.

c. Operation procedure is performed according to the instructions in the T-AOC assay kit. After the operation procedure is completed, the enzyme-labeled instrument is used to measure the absorbance of blank well, standard well and sample well at 405nm.

d. The 10mM Trolox standard solution was diluted with distilled water into 0.1, 0.2, 0.4, 0.8 and 1.0mM concentrations. The enzyme-labeled instrument is used to measure the absorbance of the standard solution with different concentrations at 405nm. The standard curve is made to calculate the T-AOC activity in each sample.

Analysis of ROS levels

a. The lower part of the fresh lung graft was embedded in an optimal cutting temperature compound and quick-frozen in liquid nitrogen.

b. The 8μm sections were prepared.

c. After washed with PBS, the sections were incubated with dihydroethidium (No.S0063, Beyotime) at 37 ℃ for 30 min in the dark.

d. Images were taken with a fluorescence inverted microscope (Olympus IX71,

Japan).

Measurement of cell necrosis marker levels

The levels of HMGB-1 in serum and BALF were detected using an enzyme-linked immunosorbent assay kit (Jingmei Biotechnology, China) according to the manufacturer's instructions. The measure methods of cell necrosis marker levels are same as inflammatory cytokine levels.

Western blot analysis

Total proteins were extracted from lung tissue homogenates using RIPA lysis buffer (Beyotime, China). The total protein concentrations were measured using an enhanced BCA protein assay kit (Beyotime, China) according to the manufacturer's instructions. Subsequently, the extracted protein samples were separated on 10–12.5% sodium dodecyl sulfate-polyacrylamide gel electrophoresis (SDS-PAGE). After electrophoresis, the proteins were transferred to polyvinylidene fluoride (PVDF) membranes, and the PVDF membranes were blocked with 5% fat-free milk for 2 h. Then, the membranes were incubated with primary antibodies against rabbit polyclonal AMPK (1:1000, AF6423, Affinity Biosciences), rabbit polyclonal p-AMPK (1:1000, AF3423, Affinity Biosciences), rabbit polyclonal RIPK1 (1:1000, AF7877, Affinity Biosciences), rabbit polyclonal RIPK3 (1:1000, AF7942, Affinity Biosciences), rabbit polyclonal MLKL (1:1000, DF7412, Affinity Biosciences), rabbit polyclonal HMGB-1 (1:1000, AF7020, Affinity Biosciences) and rabbit polyclonal β-actin (1:1000, AF7018, Affinity Biosciences) at 4℃ for 24 h. After five washes, the membranes were incubated with secondary antibody (1:1000, SA00001-2,

Proteintech) for 60 min. Finally, the immune complexes were visualized with BeyoECL Plus (Beyotime, China), and the bands were quantified with ImageJ software.
